# Detection of G-quadruplex DNA in mammalian cells

**DOI:** 10.1093/nar/gkx300

**Published:** 2017-04-25

**Authors:** Alexander Henderson, Yuliang Wu, Yu Chuan Huang, Elizabeth A. Chavez, Jesse Platt, F. Brad Johnson, Robert M. Brosh, Dipankar Sen, Peter M. Lansdorp

**Affiliations:** 1 Terry Fox Laboratory, British Columbia Cancer Agency, Vancouver, BC V5Z 1L3, Canada; 2 Laboratory of Molecular Gerontology, National Institute on Aging, National Institutes of Health, NIH Biomedical Research Center, Baltimore, MD 21224, USA; 3 Department of Chemistry, Simon Fraser University, Burnaby, BC V5A 1S6, Canada; 4 Department of Pathology and Laboratory Medicine, University of Pennsylvania, Philadelphia, PA 19104-6100, USA; 5 Division of Hematology, Department of Medicine, University of British Columbia, Vancouver, BC V6T 1Z4, Canada; 6 European Research Institute for the Biology of Ageing, University of Groningen, University Medical Centre Groningen, A. Deusinglaan 1, NL-9713 AV Groningen, The Netherlands

*Nucleic Acids Res. 2014 Jan; 42(2): 860–869. doi: 10.1093/nar/gkt957*

The corresponding Author and Editors wish to jointly express a note of concern regarding the above article.

Recent studies by the corresponding Author and new collaborators have revealed that mouse monoclonal antibody 1H6, used in the above article to detect G-quadruplexes, cross-reacts with some other DNA sequences, notably adjacent thymidines in single stranded DNA that are restricted in their movement in G4 structures and denatured DNA fibers. While the data reported in the published article remain valid, the Editors and corresponding Author wish to alert Readers of this cross-reactivity as it is likely to affect the interpretation of the results of all experiments using the 1H6 antibody.

The Editors commend the corresponding Author for being forthcoming and disclosing these latest results which have been also been published in NAR (1).

Keith Fox, Senior Executive Editor, *Nucleic Acids Research*

Barry Stoddard, Senior Executive Editor, *Nucleic Acids Research*
